# Comparison of the incidence of proteinuria and changes in eGFR among febuxostat and topiroxostat users

**DOI:** 10.1007/s10157-025-02630-x

**Published:** 2025-01-29

**Authors:** Shingo Nakayama, Michihiro Satoh, Maya Toyama, Hideaki Hashimoto, Takahisa Murakami, Takuo Hirose, Taku Obara, Takefumi Mori, Hirohito Metoki

**Affiliations:** 1 Division of Nephrology and Endocrinology, Faculty of Medicine, Tohoku Medical and Pharmaceutical University, Sendai, Japan; 2 Division of Public Health, Hygiene and Epidemiology, Faculty of Medicine, Tohoku Medical and Pharmaceutical University, Sendai, Japan; 3 Department of Preventive Medicine and Epidemiology, Tohoku Medical Megabank Organization, Tohoku University, Sendai, Japan; 4 Department of Pharmacy, Tohoku Medical and Pharmaceutical University Hospital, Sendai, Japan; 5 Department of Nephrology, Self-Defense Forces Sendai Hospital, Sendai, Japan; 6 Division of Aging and Geriatric Dentistry, Department of Rehabilitation Dentistry, Tohoku University Graduate School of Dentistry, Sendai, Japan; 7 Department of Endocrinology and Applied Medical Science, Tohoku University Graduate School of Medicine, Sendai, Japan; 8 Department of Pharmaceutical Sciences, Tohoku University Hospital, Sendai, Japan; 9 Tohoku Institute for Management of Blood Pressure, Sendai, Japan

**Keywords:** Febuxostat, Topiroxostat, eGFR changes, Proteinuria, Epidemiology

## Abstract

**Background:**

Febuxostat and topiroxostat are non-purine selective xanthine oxidoreductase inhibitors commonly used for hyperuricaemia treatment in Japan. However, comparative data on the effects of febuxostat and topiroxostat on renal function and proteinuria are limited. This study compared proteinuria incidence and changes in the estimated glomerular filtration rate (eGFR) among prevalent febuxostat and topiroxostat users.

**Methods:**

We conducted a retrospective cohort study using databases provided by DeSC Healthcare, Inc. (Tokyo, Japan). We identified 17,446 individuals (11.8% women; mean age 67.4 years) with eGFR ≥ 30 mL/min/1.73 m^2^ and no history of cardiovascular disease or proteinuria at baseline. Separate analyses were performed for individuals with eGFR < 60 mL/min/1.73 m^2^ and those with eGFR ≥ 60 mL/min/1.73 m^2^. The adjusted hazard ratio (HR) for proteinuria incidence in topiroxostat users compared with febuxostat users was assessed using the Cox model. Changes in eGFR were compared between the two groups using multiple regression analysis.

**Results:**

During the mean follow-up period of 1.79 years, 1,433 participants developed proteinuria. In non-diabetic individuals with eGFR ≥ 60 mL/min/1.73 m^2^, the adjusted HR for proteinuria incidence in topiroxostat users compared with febuxostat users was 0.60 (95% confidence interval, 0.40–0.91; *p* = 0.016). No significant differences were observed in eGFR changes between the two groups with eGFR < 60 and ≥ 60 mL/min/1.73 m^2^.

**Conclusion:**

Topiroxostat prevalent users had a lower risk of proteinuria than febuxostat prevalent users in non-diabetic individuals with eGFR ≥ 60 mL/min/1.73 m^2^. Our findings suggest that topiroxostat might be more effective than febuxostat in preventing proteinuria in non-diabetic individuals with eGFR ≥ 60 mL/min/1.73 m^2^.

**Supplementary Information:**

The online version contains supplementary material available at 10.1007/s10157-025-02630-x.

## Introduction

Chronic kidney disease (CKD) incidence, as well as disabilities and deaths due to CKD, have been increasing globally [1]. High serum uric acid levels increase the risk of CKD [2–6]. Allopurinol, febuxostat, and topiroxostat are xanthine oxidoreductase (XOR) inhibitors commonly used for the treatment of hyperuricaemia in Japan. In particular, febuxostat and topiroxostat are non-purine selective XOR inhibitors.

The Febuxostat for Cerebral and CaRdiorenovascular Events PrEvEntion StuDy (FREED) was a multicentre randomised controlled study that showed that the risk of renal events [7] or developing macroalbuminuria [8] was significantly lower in the febuxostat group than in the non-febuxostat group. In a meta-analysis of 10 studies, topiroxostat significantly improved the estimated glomerular filtration rate (eGFR) and reduced the urinary albumin/creatinine ratio (UACR) compared to the placebo in patients with CKD [9]. Previous randomised controlled trials have demonstrated that both febuxostat and topiroxostat decreased urinary albumin levels in patients with stage 3 CKD and hyperuricaemia [10, 11]. These studies suggest that febuxostat and topiroxostat may have renoprotective effects.

Several studies have compared the renoprotective effects of XOR inhibitors [12–14]. However, comparative data regarding the effects of febuxostat and topiroxostat on renal function and proteinuria are limited. Moreover, studies comparing the incidence of proteinuria and eGFR changes among febuxostat and topiroxostat users in large cohorts based on real-world data, which are used to demonstrate the effectiveness of a drug, are lacking. Therefore, this study aimed to compare the incidence of proteinuria and eGFR changes among prevalent users of febuxostat and topiroxostat based on large-scale health checkup data in Japan.

## Methods

### Study design and population

We conducted a retrospective cohort study using health insurance claims and medical checkup data collected between October 2014 and July 2021 by DeSC Healthcare, Inc. (Tokyo, Japan). The data consisted of anonymised medical information gathered by three Japanese public health insurers. Three types of insurers were included: (1) society-managed, employment-based health insurance association (provided for employees of Japanese companies and their families); (2) national health insurance (provided for individuals < 75 years old who are not covered by other public health insurance); and (3) medical care system for elderly in the latter stage of life (provided for individuals ≥ 75 years old).

**Fig. 1:**
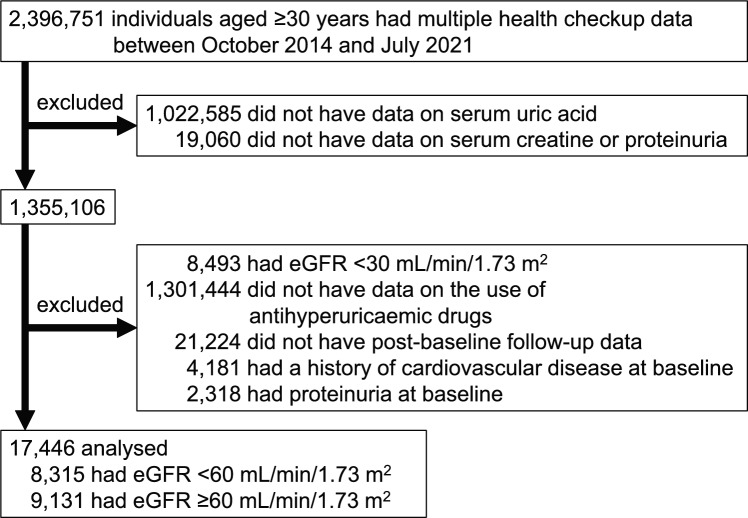
Flow chart of participants. *eGFR, estimated glomerular filtration rate*

A flowchart of the participants is shown in Fig. 1. We selected 2,396,751 individuals ≥ 30 years old with multiple health checkup data collected between October 2014 and July 2021. The measurement of serum uric acid and serum creatinine (SCr) levels is not mandatory in Japan, and 1,041,645 individuals with no data on serum uric acid or SCr levels or proteinuria were excluded. Individuals with eGFR < 30 mL/min/1.73 m^2^ may have a higher frequency of medical visits, which could reduce the likelihood of receiving health checkups. Moreover, individuals with a history of cardiovascular disease at baseline might be disproportionately more affected by the underlying disease. Therefore, we excluded individuals with eGFR < 30 mL/min/1.73 m^2^ and a history of cardiovascular disease at baseline in the present study. As a result, 1,309,937 individuals with eGFR < 30 mL/min/1.73 m^2^ and no data on urate-lowering therapy were excluded. We also excluded 21,224 patients who did not have post-baseline follow-up data. Furthermore, we excluded 4,181 individuals with a history of cardiovascular disease at baseline and 2,318 individuals with proteinuria at baseline. Eventually, the data of 17,446 participants (8,315 with eGFR < 60 mL/min/1.73 m^2^ and 9,131 with eGFR ≥ 60 mL/min/1.73 m^2^) were analysed.

### Drug data

The claims database included all inpatient, outpatient, and pharmacy claims received from insurers during the study period [15]. Prescription drug claims included the dispensing and prescription dates. The dispensing date was preferentially considered the date on which the patients received the corresponding drug rather than the prescription date. Claims contained data on prescribed medications classified by generic names and Anatomical Therapeutic Chemical codes. Antihypertensive drugs were classified into angiotensin-converting enzyme inhibitors (ACEIs), angiotensin II receptor blockers (ARBs), dihydropyridine calcium channel blockers (CCBs), benzothiazepine CCBs, thiazide diuretics (including thiazide-like diuretics), aldosterone antagonists, α-blockers, β-/αβ-blockers, and loop diuretics. If ≥ two classes of drugs were prescribed to a single patient during the same month, this was considered combination therapy. Fixed-dose combination drugs were separated based on their pharmacological components.

### Outcome data

Proteinuria was evaluated using the dipstick test for spot urine; the results were considered positive when the dipstick test value was 1 + or more, which corresponded to a urinary protein level of > 30 mg/dL. We evaluated renal function based on the eGFR, which was calculated using the modified Japanese equation involving the SCr level: eGFR (mL/min/1.73 m^2^) = 194 × (SCr in enzymatic method)^−1.094^ × Age^−0.287^ (× 0.739, if female) [16]. For the sensitivity analysis, we also calculated the eGFR based on the Chronic Kidney Disease Epidemiology Collaboration (CKD-EPI) equation modified for the Japanese population using the Japanese coefficient, as follows: eGFR_CKD-EPI_ (mL/min/1.73 m^2^) = 141 × min (Cr/κ, 1)^α^ × max (Cr/κ, 1)^−1.209^ × 0.993^Age^ × 1.018 (if female) × 0.813 (Japanese coefficient). κ and α are 0.7 and − 0.329 in females and 0.9 and − 0.411 in males, respectively; min indicates the minimum SCr/κ or 1; and max represents the maximum SCr/κ or 1 [17, 18].

### Collection of other data

The data were obtained at annual health checkups, which are recommended in the guidelines of the Japanese Ministry of Health, Labour and Welfare [19]. We gathered information on smoking status; alcohol consumption; use of antihyperuricaemic, antihypertensive, antidiabetic, and antidyslipidaemic drugs; and history of cerebrovascular disease and ischaemic heart disease through a self-administered questionnaire or interview. Hypertension was defined as systolic blood pressure ≥ 140 mmHg, diastolic blood pressure ≥ 90 mmHg, or use of antihypertensive medication. Diabetes mellitus was defined as fasting glucose level ≥ 7.00 mmol/L (≥ 126 mg/dL), random glucose level ≥ 11.11 mmol/L (≥ 200 mg/dL), HbA1c level ≥ 6.5%, or use of antidiabetic medication. Dyslipidaemia was defined as low-density lipoprotein cholesterol level ≥ 3.62 mmol/L (≥ 140 mg/dL), high-density lipoprotein cholesterol level < 1.03 mmol/L (40 mg/dL), triglyceride level ≥ 1.69 (150 mg/dL), or use of antidyslipidaemic medication.

### Follow-up and outcomes

Data from the first annual health checkup were considered the baseline data in our study. The date of proteinuria incidence was defined as the midpoint between the most recent date when the individual did not have proteinuria and the date when proteinuria was first confirmed [2, 20–22]. For participants without proteinuria, the final follow-up date was the date of the final annual health checkup in the present data. Therefore, the follow-up period was defined as the period from baseline to the date of proteinuria incidence or final checkup.

### Statistical analysis

The participants were classified according to renal function (eGFR < 60 and ≥ 60 mL/min/1.73 m^2^) since CKD is defined as the presence of kidney damage or GFR < 60 mL/min/1.73 m^2^ for ≥ 3 months in the current guideline (2012 Kidney Disease: Improving Global Outcomes (KDIGO) Clinical Practice Guideline for the Evaluation and Management of CKD) [23]. We used the standardised mean difference (SMD) to compare the means and proportions of baseline characteristics between the febuxostat and topiroxostat groups.

Using the Cox proportional hazards model, we assessed the adjusted hazard ratio (HR) for the incidence of proteinuria in the topiroxostat group compared with the febuxostat group as a reference. We performed stratified analyses according to sex, age (< 75 and ≥ 75 years), body mass index (BMI) (< 25 and ≥ 25 kg/m^2^), hypertension, and diabetes mellitus.

We calculated the annual rate of eGFR change as the slope of the linear regression between eGFR and the year for each participant. Then, only the participants with ≥ three follow-up points of eGFR data were included in the analysis to capture the slope of eGFR change as accurately as possible. Changes in eGFR were compared between the febuxostat and topiroxostat groups using multiple regression analysis.

Covariates included sex; age; BMI; current smoking status; current drinking status; diabetes mellitus; dyslipidaemia; systolic blood pressure; use of ACEIs, ARBs, CCBs, or other antihypertensive drugs; serum uric acid level; and baseline eGFR. The adjusted HRs for the incidence of proteinuria and eGFR changes were determined after adjusting for covariates and the administration of ACEIs, ARBs, or CCBs during follow-up. We further evaluated the HRs for the incidence of proteinuria excluding individuals with a follow-up period < 1 year. We assessed the changes in eGFR after excluding individuals using sodium-glucose cotransporter 2 (SGLT2) inhibitors at baseline or during follow-up, as SGLT2 inhibitors can affect changes in the eGFR. To reduce confounding bias, propensity scores for 1:4 matching of the topiroxostat and febuxostat groups were employed, using the nearest neighbour method with a calliper width set at 0.2. The propensity score was based on the following covariates: sex, age, BMI, current smoking status, current drinking status, diabetes mellitus, dyslipidaemia, systolic blood pressure, use of ACEIs, ARBs, CCBs, or other antihypertensive drugs, serum uric acid level, and baseline eGFR. All statistical analyses were performed using the SAS software (version 9.4; SAS Institute, Cary, NC, USA). Statistical significance was set at p < 0.05.

## Results

### Baseline characteristics

The mean age of the participants was 67.4 years, and 11.8% were women. The baseline characteristics of the febuxostat and topiroxostat groups with eGFR < 60 and ≥ 60 mL/min/1.73 m^2^ are listed in Table 1. SMD values ≥|0.1| between the febuxostat and topiroxostat groups were observed for age, diabetes mellitus, and uric acid level in individuals with eGFR < 60 mL/min/1.73 m^2^, and for age and uric acid level in individuals with eGFR ≥ 60 mL/min/1.73 m^2^. The baseline characteristics of patients with eGFR < 60 and ≥ 60 mL/min/1.73 m^2^ after propensity score matching are presented in Supplementary Table 1. No significant differences were observed in any variable.

**Table 1:** Baseline characteristics of the febuxostat and topiroxostat groups with eGFR < 60 and ≥ 60 mL/min/1.73 m^2^

Characteristics	Febuxostat	Topiroxostat	SMD
eGFR < 60 mL/min/1.73 m^2^			
N	7634	681	
Women, %	16.4	20.1	0.096
Age, years	71.8 ± 9.3	73.3 ± 8.8	0.16
BMI, kg/m^2^	24.5 ± 3.2	24.4 ± 3.3	− 0.022
Current smoker, %	11.9	9.5	− 0.080
Current drinker, %	37.3	37.2	− 0.0018
SBP, mmHg	131.6 ± 15.8	131.4 ± 15.4	− 0.0080
DBP, mmHg	75.9 ± 10.9	75.4 ± 10.4	− 0.054
Hypertension, %	67.1	67.3	0.0023
Dyslipidaemia, %	67.3	65.2	− 0.044
Diabetes mellitus, %	18.2	22.8	0.11
Uric acid level, mg/dL	6.1 ± 1.3	6.4 ± 1.4	0.24
SCr level, mg/dL	1.1 ± 0.18	1.1 ± 0.18	− 0.025
eGFR, mL/min/1.73 m^2^	49.4 ± 7.5	48.8 ± 7.5	− 0.082
eGFR ≥ 60 mL/min/1.73 m^2^			
N	8412	719	
Women, %	6.7	8.2	0.056
Age, years	62.4 ± 11.3	63.8 ± 11.9	0.12
BMI, kg/m^2^	25.0 ± 3.6	24.9 ± 3.9	− 0.026
Current smoker, %	21.5	23.8	0.056
Current drinker, %	51.4	52.9	0.031
SBP, mmHg	131.2 ± 15.7	132.5 ± 15.5	0.078
DBP, mmHg	78.9 ± 10.7	78.9 ± 11.5	0.0020
Hypertension, %	59.3	60.6	0.028
Dyslipidaemia, %	67.0	65.2	− 0.037
Diabetes mellitus, %	14.6	15.3	0.021
Uric acid level, mg/dL	6.1 ± 1.3	6.5 ± 1.3	0.26
SCr level, mg/dL	0.83 ± 0.11	0.83 ± 0.11	0.017
eGFR, mL/min/1.73 m^2^	73.2 ± 10.7	72.3 ± 10.8	− 0.087

*SMD* standardised mean difference, *BMI* body mass index, *SBP* systolic blood pressure, *DBP* diastolic blood pressure, *SCr* serum creatinine, *eGFR* estimated glomerular filtration rate

### Proteinuria incidence and multivariate-adjusted analysis

**Fig. 2:**
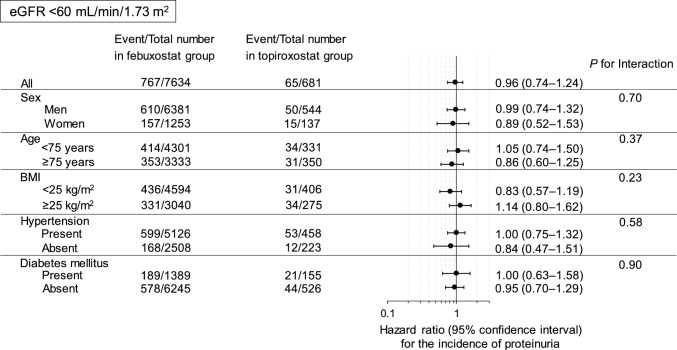
For individuals with eGFR < 60 mL/min/1.73 m^2^, we estimated the adjusted HRs (95% CI) for the incidence of proteinuria in the topiroxostat group compared with the febuxostat group using the Cox proportional hazards model. Covariates were sex, age, BMI, current smoking status, current drinking status, diabetes mellitus, dyslipidaemia, systolic blood pressure, use of ACEIs, ARBs, CCBs, or other antihypertensive drugs, serum uric acid level, and baseline eGFR. *HR* hazard ratio, *CI* confidence interval, *BMI* body mass index, *ACEI* angiotensin-converting enzyme inhibitor, *ARB* angiotensin II receptor blocker, *CCB* calcium channel blocker, *eGFR* estimated glomerular filtration rate

**Fig. 3:**
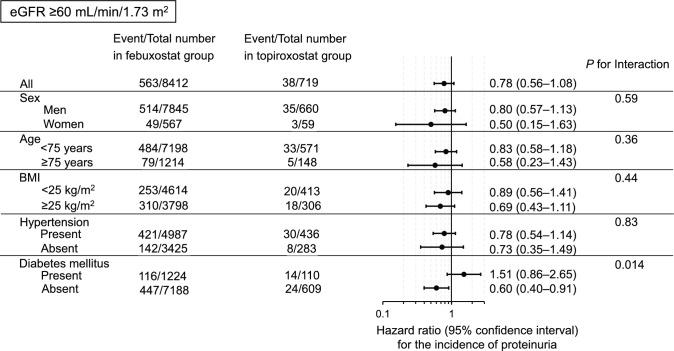
For individuals with eGFR ≥ 60 mL/min/1.73 m^2^, we estimated the adjusted HRs (95% CI) for the incidence of proteinuria in the topiroxostat group compared with the febuxostat group using the Cox proportional hazards model. Covariates and abbreviations are the same as those listed in Fig. 2

During a mean follow-up of 1.79 years (median, 1.41 years; interquartile range, 1.00–2.32 years), 1,433 participants developed proteinuria. In non-diabetic individuals with eGFR ≥ 60 mL/min/1.73 m^2^, the adjusted HR (95% confidence interval, *p*-value) for the incidence of proteinuria in the topiroxostat group compared with the febuxostat group was 0.60 (0.40–0.91, *p* = 0.016) (Figs. 2, 3). We found a significant interaction between diabetes mellitus and the two groups on the incidence of proteinuria. Similar results were observed when ACEI, ARB, or CCB administration during follow-up was added to the adjusted model (Supplementary Tables 2 and 3). After propensity score matching, similar results were also obtained (Supplementary Tables 4 and 5). Moreover, a similar tendency was observed after we excluded the individuals with a follow-up period < 1 year, although the adjusted HR for the incidence of proteinuria in the topiroxostat group compared with the febuxostat group was 0.51 (0.25–1.03, *p* = 0.061) in non-diabetic individuals with eGFR ≥ 60 mL/min/1.73 m^2^ and the significance disappeared (Supplementary Tables 6 and 7).

### Changes in eGFR in stratified or multivariate-adjusted analysis

No significant differences were observed in eGFR changes between the two groups with eGFR < 60 and ≥ 60 mL/min/1.73 m^2^ (Tables 2, 3). In the stratified analyses according to sex, age, BMI, hypertension, and diabetes mellitus, we found significant interactions for eGFR change between diabetes mellitus and the two groups with eGFR < 60 mL/min/1.73 m^2^. After adjustment for covariates with ACEI, ARB, or CCB administration during follow-up, similar results for eGFR changes were observed between the two groups with eGFR < 60 and ≥ 60 mL/min/1.73 m^2^ (Tables 2, 3). Moreover, analysis excluding individuals using SGLT2 inhibitors yielded similar results (Supplementary Tables 8 and 9). Similar results were also obtained by applying propensity score matching (Supplementary Tables 10 and 11). When we compared eGFR changes using the eGFR_CKD-EPI_, we found no significant differences in eGFR changes between the two groups with eGFR < 60 and ≥ 60 mL/min/1.73 m^2^ (Supplementary Tables 12 and 13).

**Table 2:** Changes in eGFR per year from the baseline value in the febuxostat and topiroxostat groups with eGFR < 60 mL/min/1.73 m^2^

Strata	Number in febuxostat group	Number in topiroxostat group	eGFR changes in febuxostat group	eGFR changes in topiroxostat group	Model 1		Model 2	
					*p*	*p* for interaction	*p*	*p* for interaction
All	2861	200	− 0.40 ± 4.92	− 0.28 ± 4.98	0.97		0.87	
Sex						0.064		0.057
Men	2544	175	− 0.36 ± 4.80	− 0.46 ± 4.91	0.52		0.58	
Women	317	25	− 0.71 ± 5.73	0.95 ± 5.39	0.12		0.10	
Age						0.63		0.62
< 75 years	2535	174	− 0.29 ± 4.89	− 0.11 ± 5.21	0.84		0.74	
≥ 75 years	326	26	− 1.24 ± 5.02	− 1.45 ± 2.78	0.98		0.89	
BMI						0.67		0.56
< 25 kg/m^2^	1674	116	− 0.44 ± 4.93	− 0.38 ± 5.46	0.81		0.80	
> 25 kg/m^2^	1187	84	− 0.34 ± 4.90	− 0.15 ± 4.26	0.85		0.81	
Hypertension						0.92		0.91
Present	1840	131	− 0.55 ± 5.16	− 0.41 ± 5.11	0.93		0.84	
Absent	1021	69	− 0.13 ± 4.42	− 0.03 ± 4.75	0.86		0.89	
Diabetes mellitus						0.038		0.042
Present	443	43	− 0.64 ± 6.41	1.06 ± 4.52	0.23		0.26	
Absent	2418	157	− 0.36 ± 4.59	− 0.65 ± 5.05	0.32		0.41	

We excluded from this analysis 6374 participants with two eGFR values during follow-up. Model 1 was adjusted for sex; age; BMI; current smoking status; current drinking status; diabetes mellitus; dyslipidaemia; systolic blood pressure; use of ACEIs, ARBs, CCBs, or other antihypertensive drugs; serum uric acid level; and baseline eGFR. Model 2 was adjusted for the covariates included in Model 1 in addition to ACEI, ARB, or CCB administration during follow-up

*eGFR* estimated glomerular filtration rate, *BMI* body mass index, *ACEI* angiotensin-converting enzyme inhibitor, *ARB* angiotensin II receptor blocker, *CCB*calcium channel blocker

**Table 3:** Changes in eGFR per year from the baseline value in the febuxostat and topiroxostat groups with eGFR ≥ 60 mL/min/1.73 m^2^

Strata	Number in febuxostat group	Number in topiroxostat group	eGFR changes in febuxostat group	eGFR changes in topiroxostat group	Model 1		Model 2	
					*p*	*p* for interaction	*p*	*p* for interaction
All	4315	307	− 1.46 ± 4.42	− 1.38 ± 4.70	0.96		0.92	
Sex						0.41		0.41
Men	4084	285	− 1.43 ± 4.40	− 1.30 ± 4.81	0.78		0.74	
Women	231	22	− 1.85 ± 4.74	− 2.41 ± 2.78	0.42		0.42	
Age						0.42		0.37
< 75 years	4193	298	− 1.46 ± 4.40	− 1.42 ± 4.73	0.96		0.98	
≥ 75 years	122	9	− 1.31 ± 4.80	− 0.17 ± 3.46	0.51		0.43	
BMI						0.62		0.67
< 25 kg/m^2^	2346	172	− 1.56 ± 4.31	− 1.53 ± 4.06	0.73		0.82	
> 25 kg/m^2^	1969	135	− 1.33 ± 4.54	− 1.19 ± 5.41	0.70		0.70	
Hypertension						0.44		0.43
Present	2461	194	− 1.57 ± 4.72	− 1.55 ± 5.08	0.67		0.68	
Absent	1854	113	− 1.31 ± 3.97	− 1.09 ± 3.95	0.43		0.42	
Diabetes mellitus						0.36		0.37
Present	563	37	− 1.35 ± 4.68	− 0.51 ± 5.40	0.35		0.37	
Absent	3752	270	− 1.47 ± 4.37	− 1.50 ± 4.59	0.74		0.77	

We excluded from this analysis 4921 participants with two eGFR values during follow-up. Model 1 was adjusted for sex; age; BMI; current smoking status; current drinking status; diabetes mellitus; dyslipidaemia; systolic blood pressure; use of ACEIs, ARBs, CCBs, or other antihypertensive drugs; serum uric acid level; and eGFR at baseline. Model 2 was adjusted for the covariates included in Model 1 in addition to ACEI, ARB, or CCB administration during follow-up

*eGFR* estimated glomerular filtration rate, *BMI* body mass index, *ACEI* angiotensin-converting enzyme inhibitor, *ARB* angiotensin II receptor blocker, *CCB* calcium channel blocker

## Discussion

In the present retrospective cohort study, the incidence of proteinuria and eGFR changes were compared among the prevalent users of febuxostat and topiroxostat. In the present study, the risk of proteinuria was significantly lower in the topiroxostat group than in the febuxostat group after adjusting for several covariates in non-diabetic individuals with eGFR ≥ 60 mL/min/1.73 m^2^. However, no significant differences were observed in eGFR changes between febuxostat and topiroxostat users with eGFR < 60 and ≥ 60 mL/min/1.73 m^2^. In addition, we found no significant differences between the two groups with eGFR < 60 and ≥ 60 mL/min/1.73 m^2^ in the stratified analyses according to sex, age, BMI, hypertension, and diabetes mellitus.

Our findings suggest that topiroxostat might be more effective than febuxostat in preventing proteinuria in non-diabetic individuals with eGFR ≥ 60 mL/min/1.73 m^2^. Previous studies have compared renal function and proteinuria incidence among febuxostat and allopurinol users; however, few studies have compared renal function and proteinuria incidence among febuxostat and topiroxostat users. Sezai et al. reported that febuxostat significantly improved urinary albumin levels and renal function compared with allopurinol in individuals with hyperuricaemia undergoing cardiac surgery [14]. In a randomised controlled study of a small cohort (135 patients) with both hyperuricaemia and hypertension, a significant reduction in the UACR from baseline was noted at 24 weeks in the topiroxostat group but not in the febuxostat group [12]. However, a stratification analysis according to diabetes status and baseline eGFR levels was not conducted in the aforementioned study. The long-term effects of febuxostat or topiroxostat therapy could be assessed in the present study since we utilized the data of the prevalent users of these drugs. The current study, using a real-world database, may provide useful information for the selection of XOR inhibitors in clinical practice.

Animal experiments showed that febuxostat and topiroxostat significantly reduced the UACR, and topiroxostat remarkably reduced the UACR compared to febuxostat in diabetic mice [24]. In humans, the impact of diabetic nephropathy was potent in individuals with diabetes, potentially masking the renoprotective effects of topiroxostat. In addition, urinary albumin does not correlate with changes in plasma uric acid or plasma drug concentration. Notably, topiroxostat, but not febuxostat, exhibited a correlation between urinary albumin and XOR activity in plasma [24]. Topiroxostat may decrease the UACR by inhibiting plasma XOR activity and subsequently reducing oxidative stress [24]. This mechanism could explain the relationship between topiroxostat and proteinuria incidence in our study.

The present study demonstrated no significant difference in eGFR changes between febuxostat and topiroxostat users with eGFR < 60 and ≥ 60 mL/min/1.73 m^2^, and similar results were observed even after adjusting for the administration of antihypertensive treatments at baseline or during follow-up. This may be supported by a previous study, which indicated no significant differences in the changes in eGFR from baseline at 12 and 24 weeks between the febuxostat and topiroxostat groups [12]. Meanwhile, a previous cohort study demonstrated that the proportion of patients with hyperuricaemia and stage 2–3 CKD showing a ≥ 10% decline in eGFR from baseline was smaller in the febuxostat group than in the allopurinol group [13].

It is unclear why topiroxostat only prevented proteinuria or albuminuria in the present study. Proteinuria is recognised as an early marker of kidney damage [23, 25, 26] and the incidence of proteinuria can precede changes in eGFR. Therefore, the results regarding eGFR changes might have differed with a longer follow-up period. Moreover, short-term eGFR changes may be altered by factors such as hyperfiltration.

Proteinuria not only reflects glomerular damage but also serves as an indicator of vascular damage in ‘strain vessels’, including perforating arteries and juxtamedullary afferent arterioles [27]. In patients with CKD, the use of XOR inhibitors was associated with a lower incidence of cardiovascular events [28, 29]. Therefore, the results of the present study suggest that topiroxostat may offer superior protective effects on not only the kidneys but also the vascular system compared to febuxostat.

The present study has several limitations. First, because the present study only included Japanese participants, our results may not be generalizable to other ethnic groups. Further studies are required to confirm the generalizability of our results. Second, the mean follow-up period in our study was 1.79 years, which was too short to evaluate the incidence of proteinuria and eGFR changes. The effects of febuxostat and topiroxostat on the incidence of proteinuria and eGFR changes need to be confirmed in further studies with longer follow-up periods. Third, the present study might have been affected by prevalent user bias [30]. The number of patients in the topiroxostat group was too small for accurate estimation when the present analysis was performed using only new users. Therefore, the present study did not evaluate the changes in uric acid levels resulting from the use of febuxostat and topiroxostat Given that the prevalent users of topiroxostat may have been taking the treatment for some time before the baseline, our study may indicate the relatively long-term effects of topiroxostat in clinical settings. Fourth, proteinuria was assessed through qualitative judgement using a single spot urine. However, the same criteria were applied in previous epidemiological studies [2, 20–22]. Evaluating proteinuria using consecutive positive proteinuria tests in the present study was challenging owing to the insufficient number of participants for accurate estimation.

In conclusion, topiroxostat was associated with a lower risk of proteinuria than febuxostat in non-diabetic individuals with eGFR ≥ 60 mL/min/1.73 m^2^ in our study. Our study provides useful information for the selection of XOR inhibitors in clinical settings.

## Supplementary Information

Below is the link to the electronic supplementary material.

Supplementary file1 (DOCX 111 KB)

## Data Availability

To comply with our contract with DeSC Healthcare, Inc., the data and materials from this study will not be available to other researchers.
