# Supplementary material for: Comparison of the incidence of proteinuria and changes in eGFR among febuxostat and topiroxostat users
Source: Clin Exp Nephrol. 2025 Jan 29;29(6):797–806. doi: 10.1007/s10157-025-02630-x (PMC12125080; doi:10.1007/s10157-025-02630-x)
Supplement: Supplementary file 1 — Supplementary file1 (DOCX 111 KB) [file 10157_2025_2630_MOESM1_ESM.docx]

**Supplementary Materials**

This online-only data supplement has been provided by the authors to provide readers with additional information about the study.

**Supplement to:**

**Comparison of the incidence of proteinuria and changes in eGFR among febuxostat and topiroxostat users**

**Supplementary Table 1.** Baseline characteristics of the febuxostat and topiroxostat groups with eGFR <60 and ≥60 mL/min/1.73 m^2^ after propensity score matching

| Characteristics | Febuxostat | Topiroxostat | SMD |
| --- | --- | --- | --- |
| eGFR <60 mL/min/1.73 m^2^ |  |  |  |
| N | 2721 | 681 |  |
| Women, % | 20.5 | 20.1 | -0.011 |
| Age, years | 72.9±9.2 | 73.3±8.8 | 0.041 |
| BMI, kg/m^2^ | 24.5±3.2 | 24.4±3.3 | -0.021 |
| Current smoker, % | 12.1 | 9.5 | -0.086 |
| Current drinker, % | 37.9 | 37.2 | -0.015 |
| SBP, mmHg | 131.9±15.9 | 131.4±15.4 | -0.032 |
| DBP, mmHg | 75.4±10.8 | 75.4±10.4 | -0.0017 |
| Hypertension, % | 66.9 | 67.3 | 0.0078 |
| Dyslipidaemia, % | 66.0 | 65.2 | -0.016 |
| Diabetes mellitus, % | 21.1 | 22.8 | 0.041 |
| Uric acid level, mg/dL | 6.5±1.4 | 6.4±1.4 | -0.016 |
| SCr level, mg/dL | 1.1±0.18 | 1.1±0.18 | 0.0053 |
| eGFR, mL/min/1.73 m^2^ | 48.9±7.6 | 48.8±7.5 | -0.015 |
| eGFR ≥60 mL/min/1.73 m^2^ |  |  |  |
| N | 2870 | 719 |  |
| Women, % | 8.0 | 8.2 | 0.0083 |
| Age, years | 63.6±11.3 | 63.8±11.9 | 0.017 |
| BMI, kg/m^2^ | 24.9±3.6 | 24.9±3.9 | -0.0016 |
| Current smoker, % | 21.4 | 23.8 | 0.057 |
| Current drinker, % | 52.4 | 52.9 | 0.010 |
| SBP, mmHg | 131.7±15.6 | 132.5±15.5 | 0.049 |
| DBP, mmHg | 78.7±10.6 | 78.9±11.5 | 0.015 |
| Hypertension, % | 60.0 | 60.6 | 0.012 |
| Dyslipidaemia, % | 63.9 | 65.2 | 0.027 |
| Diabetes mellitus, % | 16.1 | 15.3 | -0.023 |
| Uric acid level, mg/dL | 6.5±1.4 | 6.5±1.3 | -0.0071 |
| SCr level, mg/dL | 0.82±0.11 | 0.83±0.11 | 0.051 |
| eGFR, mL/min/1.73 m^2^ | 72.9±10.7 | 72.3±10.8 | -0.059 |

SMD, standardised mean difference; BMI, body mass index; SBP, systolic blood pressure; DBP, diastolic blood pressure; SCr, serum creatinine; eGFR, estimated glomerular filtration rate.

**Supplementary Table 2.** HRs (95% CI) for the incidence of proteinuria in the topiroxostat group compared with the febuxostat group with eGFR <60 mL/min/1.73 m^2^ considering the administration of ACEIs, ARBs, or CCBs during follow-up as a covariate

| Strata | Event/total number  in febuxostat group | Event/total number  in topiroxostat group | HR (95% CI)  for the incidence of proteinuria | *P* | *P* for interaction |
| --- | --- | --- | --- | --- | --- |
| All | 767/7634 | 65/681 | 0.95 (0.74–1.23) | 0.68 |  |
| Sex |  |  |  |  | 0.73 |
| Men | 610/6381 | 50/544 | 0.97 (0.73–1.30) | 0.85 |  |
| Women | 157/1253 | 15/137 | 0.90 (0.53–1.53) | 0.69 |  |
| Age |  |  |  |  | 0.38 |
| <75 years | 414/4301 | 34/331 | 1.03 (0.72–1.47) | 0.87 |  |
| ≥75 years | 353/3333 | 31/350 | 0.86 (0.59–1.25) | 0.43 |  |
| BMI |  |  |  |  | 0.25 |
| <25 kg/m^2^ | 436/4594 | 31/406 | 0.82 (0.57–1.18) | 0.28 |  |
| >25 kg/m^2^ | 331/3040 | 34/275 | 1.13 (0.79–1.61) | 0.51 |  |
| Hypertension |  |  |  |  | 0.62 |
| Present | 599/5126 | 53/458 | 0.99 (0.74–1.31) | 0.92 |  |
| Absent | 168/2508 | 12/223 | 0.85 (0.47–1.53) | 0.59 |  |
| Diabetes mellitus |  |  |  |  | 0.93 |
| Present | 189/1389 | 21/155 | 0.98 (0.62–1.55) | 0.92 |  |
| Absent | 578/6245 | 44/526 | 0.94 (0.69–1.28) | 0.70 |  |

Covariates were sex; age; BMI; current smoking status; current drinking status; diabetes mellitus; dyslipidaemia; systolic blood pressure; use of ACEI, ARB, CCB, or other antihypertensive drugs; serum uric acid level; baseline eGFR; and administration of ACEIs, ARBs, or CCBs during follow-up. HR, hazard ratio; CI, confidence interval; eGFR, estimated glomerular filtration rate; BMI, body mass index; ACEI, angiotensin-converting enzyme inhibitor; ARB, angiotensin II receptor blocker; CCB, calcium channel blocker.

**Supplementary Table 3.** HRs (95% CI) for the incidence of proteinuria in the topiroxostat group compared with the febuxostat group with eGFR ≥60 mL/min/1.73 m^2^ considering the administration of ACEIs, ARBs, or CCBs during follow-up as a covariate

| Strata | Event/total number  in febuxostat group | Event/total number  in topiroxostat group | HR (95% CI) for the incidence of proteinuria | *P* | *P* for interaction |
| --- | --- | --- | --- | --- | --- |
| All | 563/8412 | 38/719 | 0.78 (0.56–1.09) | 0.15 |  |
| Sex |  |  |  |  | 0.62 |
| Men | 514/7845 | 35/660 | 0.81 (0.57–1.14) | 0.22 |  |
| Women | 49/567 | 3/59 | 0.50 (0.15–1.61) | 0.24 |  |
| Age |  |  |  |  | 0.38 |
| <75 years | 484/7198 | 33/571 | 0.83 (0.58–1.19) | 0.31 |  |
| ≥75 years | 79/1214 | 5/148 | 0.57 (0.23–1.43) | 0.23 |  |
| BMI |  |  |  |  | 0.43 |
| <25 kg/m^2^ | 253/4614 | 20/413 | 0.91 (0.57–1.44) | 0.67 |  |
| >25 kg/m^2^ | 310/3798 | 18/306 | 0.69 (0.43–1.11) | 0.12 |  |
| Hypertension |  |  |  |  | 0.84 |
| Present | 421/4987 | 30/436 | 0.79 (0.54–1.15) | 0.22 |  |
| Absent | 142/3425 | 8/283 | 0.75 (0.36–1.53) | 0.42 |  |
| Diabetes mellitus |  |  |  |  | 0.018 |
| Present | 116/1224 | 14/110 | 1.51 (0.86–2.66) | 0.15 |  |
| Absent | 447/7188 | 24/609 | 0.61 (0.41–0.93) | 0.021 |  |

Covariates and abbreviations are the same as those listed in **Supplementary Table 2**.

**Supplementary Table 4.** HRs (95% CI) for the incidence of proteinuria in the topiroxostat group compared with the febuxostat group with eGFR <60 mL/min/1.73 m^2^ after propensity score matching

| Strata | Event/total number  in febuxostat group | Event/total number  in topiroxostat group | HR (95% CI)  for the incidence of proteinuria | *P* | *P* for interaction |
| --- | --- | --- | --- | --- | --- |
| All | 292/2721 | 65/681 | 0.93 (0.71–1.21) | 0.57 |  |
| Sex |  |  |  |  | 0.83 |
| Men | 219/2162 | 50/544 | 0.94 (0.69–1.28) | 0.69 |  |
| Women | 73/559 | 15/137 | 0.87 (0.50–1.53) | 0.63 |  |
| Age |  |  |  |  | 0.62 |
| <75 years | 151/1408 | 34/331 | 0.97 (0.67–1.41) | 0.87 |  |
| ≥75 years | 141/1313 | 31/350 | 0.87 (0.59–1.29) | 0.49 |  |
| BMI |  |  |  |  | 0.20 |
| <25 kg/m^2^ | 169/1645 | 31/406 | 0.78 (0.53–1.15) | 0.21 |  |
| >25 kg/m^2^ | 123/1076 | 34/275 | 1.12 (0.76–1.63) | 0.57 |  |
| Hypertension |  |  |  |  | 0.37 |
| Present | 221/1820 | 53/458 | 0.99 (0.73–1.34) | 0.96 |  |
| Absent | 71/901 | 12/223 | 0.72 (0.39–1.33) | 0.30 |  |
| Diabetes mellitus |  |  |  |  | 0.55 |
| Present | 78/573 | 21/155 | 1.07 (0.66–1.74) | 0.78 |  |
| Absent | 214/2148 | 44/526 | 0.88 (0.63–1.21) | 0.42 |  |

Propensity scores for 1:4 matching of topiroxostat and febuxostat groups were employed, using the nearest neighbour method with a calliper width set at 0.2. The propensity score was based on the following covariates: sex, age, BMI, current smoking status, current drinking status, diabetes mellitus, dyslipidaemia, systolic blood pressure, use of ACEIs, ARBs, CCBs, or other antihypertensive drugs, serum uric acid level, and baseline eGFR. HR, hazard ratio; eGFR, estimated glomerular filtration rate; BMI, body mass index; CI, confidence interval; ACEI, angiotensin-converting enzyme inhibitor; ARB, angiotensin II receptor blocker; CCB, calcium channel blocker.

**Supplementary Table 5.** HRs (95% CI) for the incidence of proteinuria in the topiroxostat group compared with in the febuxostat group with eGFR ≥60 mL/min/1.73 m^2^ after propensity score matching

| Strata | Event/total number  in febuxostat group | Event/total number  in topiroxostat group | HR (95% CI) for the incidence of proteinuria | *P* | *P* for interaction |
| --- | --- | --- | --- | --- | --- |
| All | 205/2870 | 38/719 | 0.75 (0.53–1.07) | 0.11 |  |
| Sex |  |  |  |  | 0.34 |
| Men | 180/2641 | 35/660 | 0.80 (0.56–1.16) | 0.24 |  |
| Women | 25/229 | 3/59 | 0.40 (0.12–1.39) | 0.15 |  |
| Age |  |  |  |  | 0.64 |
| <75 years | 175/2356 | 33/571 | 0.78 (0.54–1.14) | 0.20 |  |
| ≥75 years | 30/514 | 5/148 | 0.62 (0.24–1.59) | 0.32 |  |
| BMI |  |  |  |  | 0.32 |
| <25 kg/m^2^ | 91/1596 | 20/413 | 0.90 (0.55–1.47) | 0.67 |  |
| >25 kg/m^2^ | 114/1274 | 18/306 | 0.64 (0.38–1.05) | 0.078 |  |
| Hypertension |  |  |  |  | 0.73 |
| Present | 150/1723 | 30/436 | 0.78 (0.53–1.16) | 0.21 |  |
| Absent | 55/1147 | 8/283 | 0.66 (0.31–1.39) | 0.28 |  |
| Diabetes mellitus |  |  |  |  | 0.020 |
| Present | 45/463 | 14/110 | 1.49 (0.81–2.74) | 0.20 |  |
| Absent | 160/2407 | 24/609 | 0.58 (0.38–0.89) | 0.014 |  |

Propensity scores for 1:4 matching of topiroxostat and febuxostat groups were employed, using the nearest neighbour method with a calliper width set at 0.2. The propensity score was based on the following covariates: sex, age, BMI, current smoking status, current drinking status, diabetes mellitus, dyslipidaemia, systolic blood pressure, use of ACEIs, ARBs, CCBs, or other antihypertensive drugs, serum uric acid level, and baseline eGFR. HR, hazard ratio; eGFR, estimated glomerular filtration rate; BMI, body mass index; CI, confidence interval; ACEI, angiotensin-converting enzyme inhibitor; ARB, angiotensin II receptor blocker; CCB, calcium channel blocker.

**Supplementary Table 6.** HRs (95% CI) for the incidence of proteinuria in the topiroxostat group compared with the febuxostat group with eGFR <60 mL/min/1.73 m^2^ excluding individuals with a follow-up period <1 year

| Strata | Event/total number  in febuxostat group | Event/total number  in topiroxostat group | HR (95% CI)  for the incidence of proteinuria | *P* | *P* for interaction |
| --- | --- | --- | --- | --- | --- |
| All | 203/5293 | 14/459 | 0.96 (0.56–1.67) | 0.89 |  |
| Sex |  |  |  |  | 0.14 |
| Men | 165/4501 | 13/379 | 1.21 (0.69–2.15) | 0.51 |  |
| Women | 38/792 | 1/80 | 0.23 (0.031–1.74) | 0.16 |  |
| Age |  |  |  |  | 0.35 |
| <75 years | 170/3395 | 13/248 | 1.07 (0.60–1.89) | 0.83 |  |
| ≥75 years | 33/1898 | 1/211 | 0.44 (0.058–3.28) | 0.42 |  |
| BMI |  |  |  |  | 0.83 |
| <25 kg/m^2^ | 118/3187 | 7/274 | 0.89 (0.41–1.92) | 0.76 |  |
| >25 kg/m^2^ | 85/2106 | 7/185 | 1.03 (0.47–2.28) | 0.94 |  |
| Hypertension |  |  |  |  | 0.60 |
| Present | 155/3458 | 10/302 | 0.86 (0.45–1.65) | 0.66 |  |
| Absent | 48/1835 | 4/157 | 1.25 (0.44–3.54) | 0.67 |  |
| Diabetes mellitus |  |  |  |  | 0.34 |
| Present | 46/911 | 3/101 | 0.66 (0.20–2.16) | 0.49 |  |
| Absent | 157/4382 | 11/358 | 1.15 (0.62–2.13) | 0.66 |  |

We excluded 2563 individuals with a follow-up period <1 year. Covariates were sex; age; BMI; current smoking status; current drinking status; diabetes mellitus; dyslipidaemia; systolic blood pressure; use of ACEIs, ARBs, CCBs, or other antihypertensive drugs; serum uric acid level; and baseline eGFR. BMI, body mass index; HR, hazard ratio; CI, confidence interval; ACEI, angiotensin-converting enzyme inhibitor; ARB, angiotensin II receptor blocker; CCB, calcium channel blocker; eGFR, estimated glomerular filtration rate.

**Supplementary Table 7.** HRs (95% CI) for the incidence of proteinuria in the topiroxostat group compared with in the febuxostat group with eGFR ≥60 mL/min/1.73 m^2^ excluding the individuals with a follow-up period <1 year

| Strata | Event/total number  in febuxostat group | Event/total number  in topiroxostat group | HR (95% CI) for the incidence of proteinuria | *P* | *P* for interaction |
| --- | --- | --- | --- | --- | --- |
| All | 237/6591 | 10/534 | 0.54 (0.29–1.02) | 0.058 |  |
| Sex |  |  |  |  | 0.95 |
| Men | 220/6186 | 9/495 | 0.54 (0.28–1.06) | 0.071 |  |
| Women | 17/405 | 1/39 | 0.48 (0.062–3.72) | 0.48 |  |
| Age |  |  |  |  | 0.97 |
| <75 years | 226/5888 | 10/445 | 0.58 (0.31–1.10) | 0.096 |  |
| ≥75 years | 11/703 | 0/89 | – | 1.00 |  |
| BMI |  |  |  |  | 0.33 |
| <25 kg/m^2^ | 105/3642 | 6/308 | 0.79 (0.34–1.81) | 0.57 |  |
| >25 kg/m^2^ | 132/2949 | 4/226 | 0.39 (0.14–1.05) | 0.061 |  |
| Hypertension |  |  |  |  | 0.76 |
| Present | 169/3762 | 8/318 | 0.56 (0.27–1.15) | 0.11 |  |
| Absent | 68/2829 | 2/216 | 0.39 (0.094–1.66) | 0.20 |  |
| Diabetes mellitus |  |  |  |  | 0.82 |
| Present | 49/897 | 2/67 | 0.66 (0.16–2.74) | 0.57 |  |
| Absent | 188/5694 | 8/467 | 0.51 (0.25–1.03) | 0.061 |  |

We excluded 2006 individuals with a follow-up period <1 year. Covariates and abbreviations are the same as those listed in **Supplementary Table 6**.

**Supplementary Table 8.** Changes in eGFR per year from the baseline value in the febuxostat and topiroxostat groups with eGFR <60 mL/min/1.73 m^2^ after excluding the individuals using SGLT2 inhibitors at baseline or during follow-up

| Strata | Number in febuxostat group | Number in topiroxostat group | eGFR changes in febuxostat group | eGFR changes in topiroxostat group | *P* | *P* for interaction |
| --- | --- | --- | --- | --- | --- | --- |
|  |  |  |  |  |  |  |
| All | 2750 | 193 | -0.38±4.86 | -0.36±5.00 | 0.80 |  |
| Sex |  |  |  |  |  | 0.079 |
| Men | 2450 | 169 | -0.34±4.75 | -0.52±4.93 | 0.38 |  |
| Women | 300 | 24 | -0.74±5.69 | 0.76±5.43 | 0.16 |  |
| Age |  |  |  |  |  | 0.66 |
| <75 years | 2427 | 167 | -0.28±4.85 | -0.20±5.25 | 0.95 |  |
| ≥75 years | 323 | 26 | -1.19±4.92 | -1.45±2.78 | 0.99 |  |
| BMI |  |  |  |  |  | 0.58 |
| <25 kg/m^2^ | 1639 | 113 | -0.42±4.89 | -0.48±5.46 | 0.62 |  |
| >25 kg/m^2^ | 1111 | 80 | -0.34±4.83 | -0.20±4.30 | 0.95 |  |
| Hypertension |  |  |  |  |  | 0.92 |
| Present | 1762 | 128 | -0.53±5.10 | -0.53±5.09 | 0.78 |  |
| Absent | 988 | 65 | -0.13±4.40 | -0.035±4.84 | 0.89 |  |
| Diabetes mellitus |  |  |  |  |  | 0.076 |
| Present | 342 | 36 | -0.61±6.48 | 0.88±4.64 | 0.37 |  |
| Absent | 2408 | 157 | -0.35±4.59 | -0.65±5.05 | 0.32 |  |

A total of 118 individuals using SGLT2 inhibitors at baseline or during follow-up were excluded. Covariates included sex, age, BMI, current smoking status, current drinking status, diabetes mellitus, dyslipidaemia, systolic blood pressure, use of ACEIs, ARBs, CCBs, or other antihypertensive drugs, serum uric acid level, and baseline eGFR. eGFR, estimated glomerular filtration rate; SGLT2, sodium-glucose cotransporter 2; BMI, body mass index; ACEI, angiotensin-converting enzyme inhibitor; ARB, angiotensin II receptor blocker; CCB, calcium channel blocker.

**Supplementary Table 9.** Changes in eGFR per year from the baseline value in the febuxostat and topiroxostat groups with eGFR ≥60 mL/min/1.73 m^2^ after excluding the individuals using SGLT2 inhibitors at baseline or during follow-up

| Strata | Number in febuxostat group | Number in topiroxostat group | eGFR changes in febuxostat group | eGFR changes in topiroxostat group | *P* | *P* for interaction |
| --- | --- | --- | --- | --- | --- | --- |
|  |  |  |  |  |  |  |
| All | 4153 | 295 | -1.46±4.38 | -1.34±4.75 | 0.90 |  |
| Sex |  |  |  |  |  | 0.49 |
| Men | 3941 | 275 | -1.43±4.36 | -1.27±4.85 | 0.74 |  |
| Women | 212 | 20 | -1.94±4.70 | -2.41±2.92 | 0.46 |  |
| Age |  |  |  |  |  | 0.42 |
| <75 years | 4034 | 286 | -1.46±4.37 | -1.38±4.78 | 0.98 |  |
| ≥75 years | 119 | 9 | -1.29±4.85 | -0.17±3.46 | 0.49 |  |
| BMI |  |  |  |  |  | 0.50 |
| <25 kg/m^2^ | 2305 | 167 | -1.57±4.29 | -1.54±4.07 | 0.71 |  |
| >25 kg/m^2^ | 1848 | 128 | -1.31±4.49 | -1.08±5.51 | 0.60 |  |
| Hypertension |  |  |  |  |  | 0.41 |
| Present | 2365 | 185 | -1.53±4.69 | -1.49±5.15 | 0.69 |  |
| Absent | 1788 | 110 | -1.35±3.93 | -1.10±3.98 | 0.37 |  |
| Diabetes mellitus |  |  |  |  |  | 0.18 |
| Present | 422 | 28 | -1.38±4.45 | -0.015±5.81 | 0.25 |  |
| Absent | 3731 | 267 | -1.46±4.38 | -1.48±4.61 | 0.76 |  |

A total of 174 individuals using SGLT2 inhibitors at baseline or during follow-up were excluded. Covariates and abbreviations are the same as those listed in **Supplementary Table 8**.

**Supplementary Table 10.** Changes in eGFR per year from the baseline value in the febuxostat and topiroxostat groups with eGFR <60 mL/min/1.73 m^2^ after propensity score matching

| Strata | Number in febuxostat group | Number in topiroxostat group | eGFR changes in febuxostat group | eGFR changes in topiroxostat group | *P* | *P* for interaction |
| --- | --- | --- | --- | --- | --- | --- |
|  |  |  |  |  |  |  |
| All | 799 | 200 | -0.47±4.88 | -0.28±4.98 | 0.69 |  |
| Sex |  |  |  |  |  | 0.080 |
| Men | 698 | 175 | -0.39±4.65 | -0.46±4.91 | 0.84 |  |
| Women | 101 | 25 | -1.02±6.26 | 0.95±5.39 | 0.22 |  |
| Age |  |  |  |  |  | 0.93 |
| <75 years | 694 | 174 | -0.28±4.91 | -0.11±5.21 | 0.67 |  |
| ≥75 years | 105 | 26 | -1.67±4.55 | -1.45±2.78 | 0.95 |  |
| BMI |  |  |  |  |  | 0.85 |
| <25 kg/m^2^ | 466 | 116 | -0.57±4.92 | -0.38±5.46 | 0.88 |  |
| >25 kg/m^2^ | 333 | 84 | -0.32±4.83 | -0.15±4.26 | 0.83 |  |
| Hypertension |  |  |  |  |  | 0.91 |
| Present | 559 | 131 | -0.62±5.11 | -0.41±5.11 | 0.67 |  |
| Absent | 240 | 69 | -0.10±4.28 | -0.033±4.75 | 0.95 |  |
| Diabetes mellitus |  |  |  |  |  | 0.0065 |
| Present | 134 | 43 | -1.45±5.64 | 1.06±4.52 | 0.035 |  |
| Absent | 665 | 157 | -0.27±4.69 | -0.65±5.05 | 0.35 |  |

Propensity scores for 1:4 matching of topiroxostat and febuxostat groups were applied using the nearest neighbour method with a calliper width set at 0.2. The propensity score was based on the following covariates: sex, age, BMI, current smoking status, current drinking status, diabetes mellitus, dyslipidaemia, systolic blood pressure, use of ACEIs, ARBs, CCBs, or other antihypertensive drugs, serum uric acid level, and baseline eGFR. eGFR, estimated glomerular filtration rate; BMI, body mass index; ACEI, angiotensin-converting enzyme inhibitor; ARB, angiotensin II receptor blocker; CCB, calcium channel blocker.

**Supplementary Table 11.** Changes in eGFR per year from the baseline value in the febuxostat and topiroxostat groups with eGFR ≥60 mL/min/1.73 m^2^ after propensity score matching

| Strata | Number in febuxostat group | Number in topiroxostat group | eGFR changes in febuxostat group | eGFR changes in topiroxostat group | *P* | *P* for interaction |
| --- | --- | --- | --- | --- | --- | --- |
|  |  |  |  |  |  |  |
| All | 1224 | 306 | -1.33±4.61 | -1.36±4.69 | 0.74 |  |
| Sex |  |  |  |  |  | 0.37 |
| Men | 1154 | 284 | -1.33±4.53 | -1.28±4.80 | 0.89 |  |
| Women | 70 | 22 | -1.36±5.82 | -2.41±2.78 | 0.35 |  |
| Age |  |  |  |  |  | 0.83 |
| <75 years | 1173 | 297 | -1.37±4.56 | -1.40±4.72 | 0.79 |  |
| ≥75 years | 51 | 9 | -0.46±5.61 | -0.17±3.46 | 0.71 |  |
| BMI |  |  |  |  |  | 0.66 |
| <25 kg/m^2^ | 667 | 172 | -1.50±4.44 | -1.53±4.06 | 0.52 |  |
| >25 kg/m^2^ | 557 | 134 | -1.14±4.79 | -1.14±5.40 | 0.98 |  |
| Hypertension |  |  |  |  |  | 0.41 |
| Present | 718 | 193 | -1.38±4.99 | -1.52±5.08 | 0.47 |  |
| Absent | 506 | 113 | -1.27±4.01 | -1.09±3.95 | 0.66 |  |
| Diabetes mellitus |  |  |  |  |  | 0.72 |
| Present | 170 | 37 | -0.90±5.58 | -0.51±5.40 | 0.79 |  |
| Absent | 1054 | 269 | -1.40±4.43 | -1.48±4.58 | 0.63 |  |

Propensity scores for 1:4 matching of topiroxostat and febuxostat groups were applied using the nearest neighbour method with a calliper width set at 0.2. The propensity score was based on the following covariates: sex, age, BMI, current smoking status, current drinking status, diabetes mellitus, dyslipidaemia, systolic blood pressure, use of ACEIs, ARBs, CCBs, or other antihypertensive drugs, serum uric acid level, and baseline eGFR. eGFR, estimated glomerular filtration rate; BMI, body mass index; ACEI, angiotensin-converting enzyme inhibitor; ARB, angiotensin II receptor blocker; CCB, calcium channel blocker.

# **Supplementary Table 12.** Changes in eGFR per year from the baseline value in the febuxostat and topiroxostat groups with eGFR <60 mL/min/1.73 m^2^ using the eGFR_CKD-EPI_

| Strata | Number in febuxostat group | Number in topiroxostat group | eGFR changes in febuxostat group | eGFR changes in topiroxostat group | Model 1 | | Model 2 | |
| --- | --- | --- | --- | --- | --- | --- | --- | --- |
|  |  |  |  |  | *P* | *P*  for interaction | *P* | *P* for interaction |
| All | 1987 | 150 | -0.63±5.42 | -0.23±5.18 | 0.48 |  | 0.43 |  |
| Sex |  |  |  |  |  | 0.25 |  | 0.24 |
| Men | 1757 | 130 | -0.58±5.26 | -0.33±5.09 | 0.76 |  | 0.73 |  |
| Women | 230 | 20 | -1.01±6.50 | 0.42±5.89 | 0.19 |  | 0.18 |  |
| Age |  |  |  |  |  | 0.45 |  | 0.44 |
| <75 years | 1701 | 125 | -0.45±5.38 | 0.10±5.46 | 0.35 |  | 0.30 |  |
| ≥75 years | 286 | 25 | -1.67±5.53 | -1.91±3.10 | 0.97 |  | 0.90 |  |
| BMI |  |  |  |  |  | 0.76 |  | 0.60 |
| <25 kg/m^2^ | 1195 | 91 | -0.66±5.35 | -0.30±5.42 | 0.61 |  | 0.69 |  |
| >25 kg/m^2^ | 792 | 59 | -0.57±5.53 | -0.12±4.85 | 0.62 |  | 0.56 |  |
| Hypertension |  |  |  |  |  | 0.61 |  | 0.64 |
| Present | 1304 | 102 | -0.78±5.46 | -0.22±5.40 | 0.37 |  | 0.34 |  |
| Absent | 683 | 48 | -0.33±5.32 | -0.26±4.74 | 0.80 |  | 0.84 |  |
| Diabetes mellitus |  |  |  |  |  | 0.063 |  | 0.077 |
| Present | 316 | 32 | -0.95±6.62 | 1.33±5.32 | 0.15 |  | 0.16 |  |
| Absent | 1671 | 118 | -0.57±5.16 | -0.66±5.09 | 0.75 |  | 0.83 |  |

Model 1 was adjusted for sex; age; BMI; current smoking status; current drinking status; diabetes mellitus; dyslipidaemia; systolic blood pressure; use of ACEIs, ARBs, CCBs, or other antihypertensive drugs; serum uric acid level; and baseline eGFR. Model 2 was adjusted for the covariates included in Model 1 in addition to ACEI, ARB, or CCB administration during follow-up. eGFR, estimated glomerular filtration rate; CKD-EPI, Chronic Kidney Disease Epidemiology Collaboration; BMI, body mass index; ACEI, angiotensin-converting enzyme inhibitor; ARB, angiotensin II receptor blocker; CCB, calcium channel blocker.

**Supplementary Table 13.** Changes in eGFR per year from the baseline value in the febuxostat and topiroxostat groups with eGFR ≥60 mL/min/1.73 m^2^ using the eGFR_CKD-EPI_

| Strata | Number in febuxostat group | Number in topiroxostat group | eGFR changes in febuxostat group | eGFR changes in topiroxostat group | Model 1 | | Model 2 | |
| --- | --- | --- | --- | --- | --- | --- | --- | --- |
|  |  |  |  |  | *P* | *P* for interaction | *P* | *P* for interaction |
| All | 5193 | 358 | -1.49±3.39 | -1.56±3.49 | 0.72 |  | 0.77 |  |
| Sex |  |  |  |  |  | 0.58 |  | 0.58 |
| Men | 4871 | 330 | -1.49±3.38 | -1.58±3.50 | 0.60 |  | 0.64 |  |
| Women | 322 | 28 | -1.61±3.49 | -1.27±3.36 | 0.68 |  | 0.61 |  |
| Age |  |  |  |  |  | 0.35 |  | 0.31 |
| <75 years | 5031 | 348 | -1.48±3.39 | -1.58±3.52 | 0.65 |  | 0.68 |  |
| ≥75 years | 162 | 10 | -1.83±3.17 | -0.92±1.72 | 0.32 |  | 0.37 |  |
| BMI |  |  |  |  |  | 0.52 |  | 0.56 |
| <25 kg/m^2^ | 2827 | 197 | -1.55±3.27 | -1.73±3.35 | 0.44 |  | 0.50 |  |
| >25 kg/m^2^ | 2366 | 161 | -1.42±3.52 | -1.35±3.64 | 0.84 |  | 0.83 |  |
| Hypertension |  |  |  |  |  | 0.40 |  | 0.41 |
| Present | 3000 | 224 | -1.65±3.70 | -1.80±3.79 | 0.44 |  | 0.47 |  |
| Absent | 2193 | 134 | -1.27±2.89 | -1.15±2.87 | 0.58 |  | 0.59 |  |
| Diabetes mellitus |  |  |  |  |  | 0.25 |  | 0.25 |
| Present | 690 | 48 | -1.47±3.75 | -0.89±3.32 | 0.33 |  | 0.32 |  |
| Absent | 4503 | 310 | -1.50±3.33 | -1.66±3.51 | 0.43 |  | 0.46 |  |

Model 1 was adjusted for sex; age; BMI; current smoking status; current drinking status; diabetes mellitus; dyslipidaemia; systolic blood pressure; use of ACEIs, ARBs, CCBs, or other antihypertensive drugs; serum uric acid level; and eGFR at baseline. Model 2 was adjusted for the covariates included in Model 1 in addition to ACEI, ARB, or CCB administration during follow-up. eGFR, estimated glomerular filtration rate; CKD-EPI, Chronic Kidney Disease Epidemiology Collaboration; BMI, body mass index; ACEI, angiotensin-converting enzyme inhibitor; ARB, angiotensin II receptor blocker; CCB, calcium channel blocker.
